# Professional Success

**Published:** 1870-02

**Authors:** 


### Editorial Department.

### Professional Success.

As we are about to graduate from our various Colleges a large number of young men, all eager for success; and as the medical public is made up largely of young men, many of whom are often inquiring anxiously for the "philosophers stone"—the true secret of professional success—we have thought how opportune would be the announcement that it has been truly discovered. *Age* is not necessary to success. Many of our distinguished men, those who have most signally met professional prosperity are yet young in years. Friends and family influences are not important in attaining to fortunes or professional fame, the event of success, does not consist in outside and accidental circumstances, but is really the natural fruit of that inner motive which guides the conduct and controls the action. In what direction shall the physician seek his objects of ambition with any hope of gaining them ? Does it matter in what town or city ? Is there any place in the civilized world where they may not be found ? Can he extract true professional fame from political, social or church influences, or can it be drawn from any other but its true source ?

I assure young physicians, that it is within easy reach of all, and that you have only to reach for it in the right direction and it is yours. "If you make yourselves what you would have others regard you," the objeet you are seeking is gained, and it can never be reached in any other way.

We have watched the ways of the profession for nearly a quarter of a century, and we have never known any single instance where a physician attained to any high standing in the profession or community by watching jealously the growth of others, forgetting how much more important for them to preserve and increase their own reputation, than to detract from that of others. Indeed, by aiding and strengthening their associates, men grow stronger and more vigorous themselves, while by casting little hindrances in their way, they grow smaller and smaller themselves, and are finally lost in nothingness. Again, we have never known any one attain to any of the higher degrees of professional excellence, or receive any of the better rewards of professional worth, who based their claims and expectations upon anything but professional merit. If we are to be honored and respected as men, we must be upright and honorable. If we would receive the confidence and snpport of individuals and communities, but one avenue is open for its attainment. We are measured fairly at last, and at our true value.

